# Comprehensive CT Imaging Analysis of Primary Colorectal Squamous Cell Carcinoma: A Retrospective Study

**DOI:** 10.3390/tomography10050052

**Published:** 2024-05-01

**Authors:** Eun Ju Yoon, Sang Gook Song, Jin Woong Kim, Hyun Chul Kim, Hyung Joong Kim, Young Hoe Hur, Jun Hyung Hong

**Affiliations:** 1 Department of Radiology, Chosun University Hospital and Chosun University College of Medicine, Gwangju 61453, Republic of Korea; racidian@gmail.com (E.J.Y.); sgsong71@gmail.com (S.G.S.);; 2 Medical Science Research Institute, Kyung Hee University Hospital, Seoul 02447, Republic of Korea; 3 Department of Hepato-Biliary-Pancreas Surgery, Chonnam National University Hwasun Hospital and Chonnam National University Medical School, Gwangju 61469, Republic of Korea

**Keywords:** squamous-cell carcinoma, colon, rectum, computed tomography (CT)

## Abstract

The aim of this study was to evaluate the findings of CT scans in patients with pathologically confirmed primary colorectal squamous-cell carcinoma (SCC). The clinical presentation and CT findings in eight patients with pathologically confirmed primary colorectal squamous-cell carcinoma were retrospectively reviewed by two gastrointestinal radiologists. Hematochezia was the most common symptom (n = 5). The tumors were located in the rectum (n = 7) and sigmoid colon (n = 1). The tumors showed circumferential wall thickening (n = 4), bulky mass (n = 3), or eccentric wall thickening (n = 1). The mean maximal wall thickness of the involved segment was 29.1 mm ± 13.4 mm. The degree of tumoral enhancement observed via CT was well enhanced (n = 4) or moderately enhanced (n = 4). Necrosis within the tumor was found in five patients. The mean total number of metastatic lymph nodes was 3.1 ± 3.3, and the mean short diameter of the largest metastatic lymph node was 16.6 ± 5.7 mm. Necrosis within the metastatic node was observed in six patients. Invasions to adjacent organs were identified in five patients (62.5%). Distant metastasis was detected in only one patient. In summary, primary SCCs that arise from the colorectum commonly present as marked invasive wall thickening or a bulky mass with heterogeneous well-defined enhancement, internal necrosis, and large metastatic lymphadenopathies.

## 1. Background

Primary squamous-cell carcinoma (SCC) of the gastrointestinal tract most commonly occurs in the esophagus and anus. According to a report published by the World Health Organization, nearly half of the new cases of esophageal cancer worldwide in 2020 occurred in China, with 90% of them being esophageal SCC [1]. In the colorectum, most primary malignancies are adenocarcinomas, with the other rare tumors occurring in the colorectum being neuroendocrine tumors, lymphomas, leiomyosarcomas, and gastrointestinal stromal tumors [1,2]. Among the primary malignancies of the gastrointestinal tract, it is extremely rare for SCC to arise in the colorectum, and its rarity has led some authors to question its existence. However, it is known that it accounts for approximately 0.1 to 0.2% of all rectal malignancies [2,3,4,5,6].

The rarity of primary SCC originating from the colorectum has resulted in a significant dearth of comprehensive data on its epidemiology, pathogenesis, and management. The exact mechanism of its development remains unclear, although several hypotheses have been proposed. 

Although the clinical presentation of primary colorectal SCC is similar to colorectal adenocarcinoma when the tumor is first detected, primary colorectal SCC has been reported to have a poorer prognosis than adenocarcinoma [2,7,8]. In both Europe and America, the efficacy of the treatment methods utilized for anal cancer has been the focus of various prospective randomized controlled trials. These studies have consistently demonstrated the superiority of synchronous chemoradiation compared to the sole use of radiotherapy [9,10,11]. As a result of these findings, synchronous chemoradiation has been adopted as the established standard treatment protocol for SCC of the anal region. A study by Nahas et al. [12], which was conducted over 22 years, investigated the treatment of 12 rectal SCC patients and found that chemoradiation therapy (CRT) was particularly effective. Two patients achieved complete remission with CRT alone, and locoregional control was achieved in five out of the six CRT-treated patients, which parallels findings regarding anal cancer. This underscores CRT’s viability and distinctiveness as a treatment for rectal SCC compared to its efficacy for colorectal adenocarcinoma.

There is little information in the literature regarding the imaging findings for colorectal SCC [7,8,12]. In order, tumors most often occur at the following sites: rectum, right, sigmoid, transverse, descending, and proximal colon [7]. The average size of colorectal SCC is 7.9 cm (range: 4–15 cm), and SCC generally exhibits a large tumor size [7,8]. The most common sites of metastases, in order, are the liver, peritoneum, and lung [7].

It is important to note that, despite the differing clinical characteristics of the two tumor types, an accurate staging system for primary colorectal SCC and its specific imaging findings has not yet been conclusively established. Although there have been over 400 studies published on primary colorectal SCC, detailed imaging findings for SCC have so far only been described in a limited number of case reports [13]. This scarcity of information presents a significant challenge in the accurate diagnosis of primary colorectal SCC using imaging techniques. Consequently, our study aimed to analyze the records of patients with pathologically confirmed primary colorectal SCC in order to better characterize the appearance of primary colorectal SCC on CT imaging. This endeavor seeks to contribute to our understanding of the imaging findings for primary colorectal SCC, thereby aiding in the refinement of diagnostic processes for this condition.

## 2. Materials and Methods

### 2.1. Study Design

Due to the rarity of SCC, the present study necessitated an exhaustive review of patient records that extended over a span of two decades; these were collected from three prominent tertiary healthcare institutions. The methodology included the methodical gathering and subsequent detailed analysis of data specifically related to patients who had been diagnosed with SCC. The purpose of this meticulous process was to foster a deeper and more nuanced understanding of the disease in the given context. Ethical approval for this study was duly obtained from the institutional review board (IRB) of Chonnam National University Hwasun Hospital (IRB No. CNUHH-2017-139). In accordance with the IRB’s guidelines, the requirement for informed consent was waived; this was primarily due to the retrospective nature of the study, which involved the use of existing medical records.

### 2.2. Patients

We conducted an in-depth review of patient records obtained from three major tertiary healthcare institutions from January 2000 to December 2020, identifying a cohort of thirteen patients with pathologically confirmed SCC in the colorectum. However, a subset of these patients, specifically five individuals whose SCC had extended to the anus, were excluded from the study. This exclusion was based on specific diagnostic criteria for primary colorectal SCC, as outlined in a previous study [14]: (a) patients whose colon cancer had metastasized from another site were excluded; (b) patients whose squamous-lined fistula tract involved the affected bowel were excluded, as this could be a potential source of SCC; and (c) patients in whom SCC of the anus extended proximally were excluded. 

Following these criteria, the study ultimately focused on eight patients (comprising an equal gender distribution of four men and four women) who had pathologically confirmed SCC of the colorectum, which was ascertained via a surgical operation. This surgical confirmation involved six cases of surgical resection and two cases of palliative colostomy. The age range of these patients was notably varied, extending from 54 to 86 years, with a mean age of 69.3 years; the standard deviation was 13.0 years. For all these patients, comprehensive clinical records were available, encompassing both the clinical presentation and the pathological confirmation of their diagnoses. To ensure the meticulous analysis and interpretation of data, the clinical presentation of each patient was individually reviewed by one of the authors; this author thoroughly examined the clinical records to glean detailed insights into the nature and progression of the disease in each case.

### 2.3. CT Scanning

In this study, all participants underwent preoperative imaging using contrast-enhanced computed tomography (CT). The CT examinations utilized multi-channel detector CT systems of varying capacities: two patients were scanned using a 4-channel LightSpeed QX/I system, produced by GE Healthcare (Milwaukee, WI, USA), another two were scanned with a 16-channel LightSpeed 16 system, also produced by GE Healthcare (Milwaukee, WI, USA), and the remaining four patients were scanned using a 64-channel Sensation Cardiac 64 system, produced by Siemens Medical Solutions (Forchheim, Germany). The imaging parameters included a slice thickness and interval that ranged between 3.8 and 5 mm. Unenhanced CT images were specifically obtained for five of the patients. Additionally, portal venous phase images were acquired for all patients; this was following the administration of intravenous contrast material at a flow rate of 2 to 3 mL/s, which was delivered using an automatic power injector. Notably, bowel preparation was conducted in one patient, and oral Gastrografin was administered prior to the examination in a separate case. The scan coverage extended from the upper abdomen at the level of the hepatic dome down to the anal verge, specifically during the portal venous phase. Complementary imaging techniques were also employed for some patients: ultrasound was performed on two patients, and 18F-FDG PET-CT scans were conducted on another two of the eight patients, thus adding further diagnostic depth to the study.

### 2.4. Image Analysis

In this comprehensive study, two specialized gastrointestinal radiologists undertook a detailed review of the CT scans in order to reach mutual agreement regarding their findings. The aspects they meticulously evaluated are described as follows. Firstly, they pinpointed the exact location of the tumor within the colon. They then assessed the morphological tumor type, which included identifying specific features such as whether the tumor caused the circumferential or eccentric thickening of the bowel wall, or if it appeared as a bulky mass. Circumferential wall thickening was identified and defined as a condition in which the segment of the bowel affected by the tumor exhibited even thickening throughout its entire circumference. This means that the thickening was uniform all around the segment, suggesting a particular pattern of tumor growth. In contrast, eccentric wall thickening was characterized by varying degrees of thickening in different parts of the involved bowel segment [15]. This variation in thickening could indicate a different pattern of tumor growth or involvement, with some areas being more affected than others. This type of thickening is not uniform and can be more irregular compared to circumferential thickening. Additionally, a bulky mass was defined distinctly from the aforementioned types. This term referred to a lesion that was a large, noticeable tumor and markedly different from the patterns of either circumferential or eccentric wall thickening. Essentially, a bulky mass is a large tumor growth that stands out from the surrounding tissue, rather than causing the wall of the bowel to thicken in a uniform or irregular manner. 

**Figure 1:**
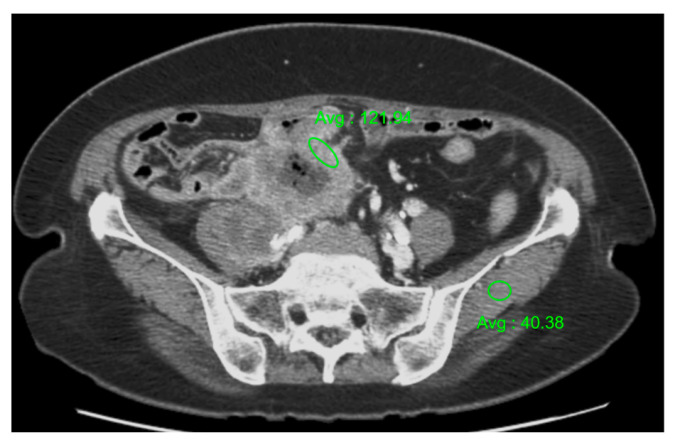
A CT image of the method used to measure the ratio of maximum contrast enhancement between the tumor and the back muscle in the same phase of a contrast-enhanced CT scan. A ratio of 3.01, as shown, is categorized as a good enhancement level (ratio > 1.5).

Further, the radiologists measured the maximum thickness of the bowel wall segment affected by the SCC. They also observed the pattern of enhancement that the tumor exhibited on the portal venous phase images of the CT scans, and determined whether the enhancement was uniform (homogeneous) or varied (heterogeneous) across the tumor. The radiologists also assessed the ratio of the maximum contrast enhancement between the tumor and the back muscle in the same phase of the contrast-enhanced CT scan (Figure 1). They categorized the enhancement levels as good (ratio > 1.5), moderate (ratio between 1 and 1.5), or poor (ratio < 1).

Additionally, the radiologists checked for the presence of necrosis within the tumor; this was identified as areas within the tumor that showed little or no enhancement. The absence of malignant bowel obstruction or signs of it were also noted; this was identified based on specific criteria, including clinical and radiographic evidence [16]. The number of metastatic lymph nodes was counted, with a lymph node considered metastatic if its short diameter exceeded 7 mm. They also measured the short diameter of the largest metastatic lymph node. In cases of lymph nodes, the absence or presence of necrosis (defined as non- or poorly enhanced areas within what were presumed to be metastatic lymph nodes) was also recorded. Finally, they evaluated whether invasion into the structures adjacent to the tumor had occurred and whether there was any evidence of distant metastasis into the abdomen. This comprehensive assessment provided a detailed understanding of the tumor’s characteristics and spread, which is crucial for the effective planning of treatment.

Regarding the diagnosis of malignant bowel obstruction, the criteria were twofold [16]. First, clinical evidence of bowel obstruction was required. This evidence could be gathered from the patient’s past medical history, physical examination findings, and radiographic examination, specifically CT scans. The CT findings that were indicative of obstruction included the dilation of the bowel segment proximal to the obstruction, the presence of a distinct transition point showing where the obstruction has occurred, and the decompression of the bowel distal to the obstruction. Second, there must be documentation of either an intra-abdominal primary cancer or a non-intra-abdominal primary cancer that has clearly spread to the intraperitoneal area. This comprehensive approach ensured that the diagnosis of malignant bowel obstruction was both accurate and reliable, taking into account a range of clinical and radiographic factors.

## 3. Results

The clinical presentation and CT findings of the eight patients with primary colorectal SCC are summarized in Table 1. The clinical symptoms that these patients presented with were diverse: hematochezia was observed in five patients; abdominal pain was reported by two patients; and one patient experienced fever. Interestingly, one patient showed symptoms of both constipation and hematochezia.

**Figure 2:**
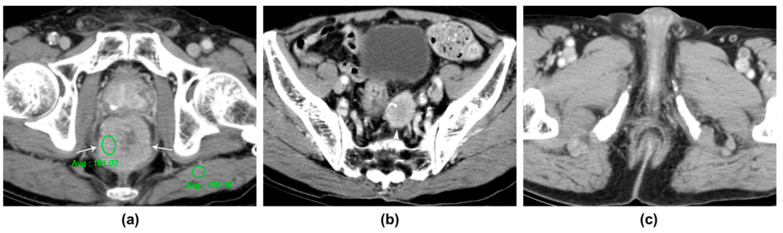
An 86-year-old man with squamous cell carcinoma of the rectum. (**a**) The contrast-enhanced portal venous phase CT image demonstrates homogeneously well-enhanced marked circumferential wall thickening (arrows) of the rectum. The ratio of tumor-to-back-muscle enhancement is 1.16 and is categorized as a moderate enhancement level (ratio between 1 and 1.5). (**b**) The second contrast-enhanced portal venous phase CT image depicts a large metastatic lymph node (arrowhead) with a spiculated margin and central necrosis (curved arrow) along the superior rectal vessel. (**c**) The third contrast-enhanced portal venous phase CT image reveals an intact anus without tumoral involvement.

**Figure 3:**
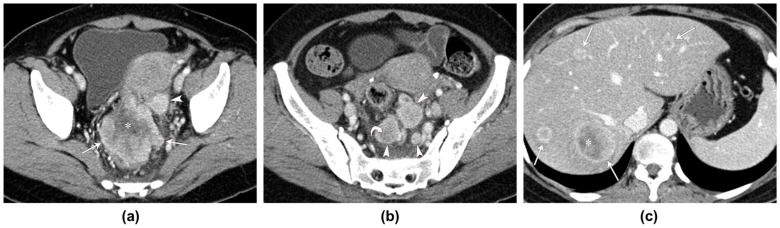
A 54-year-old woman with squamous cell carcinoma of the rectum. (**a**,**b**) Contrast-enhanced portal venous phase CT images demonstrate an infiltrative, heterogeneously well-enhanced bulky mass (arrows) in the upper rectum with central necrosis (asterisk) and invasion of the uterus. CT images also show multiple large metastatic lymph nodes (arrowheads) with some central necrosis (curved arrow). (**c**) The contrast-enhanced portal venous phase CT image depicts multiple peripheral well-enhanced metastatic nodules (arrows), with some central necrosis (asterisk) in both hepatic lobes.

Regarding the location of the tumors, the majority were found in the rectum, with seven cases identified; meanwhile, one tumor was located in the sigmoid colon. The morphological features of these tumors, as observed on the CT scans, varied: four patients showed circumferential wall thickening, three patients exhibited a bulky mass, and one demonstrated eccentric wall thickening. The imaging finding of SCC displaying eccentric wall thickening in one patient is akin to that observed in adenocarcinoma. The morphological findings of circumferential wall thickening and bulky mass are depicted in Figure 2 and Figure 3, respectively.

The impact of the tumors was further quantified by measuring the maximum thickness of the wall in the segment of the bowel affected by the tumor. The thickness varied considerably, ranging from 14 mm to as much as 49 mm, with an average thickness of approximately 29.1 mm (with a standard deviation of 13.4 mm). Regarding the enhancement patterns seen on the portal venous phase images of the CT scans, these were categorized as either homogeneous, as observed in five patients, or heterogeneous, as observed in three patients. A bowel loop with heterogeneous enhancement was identified by its stratified or mixed pattern of attenuation, as detailed in the study.

Additionally, the contrast enhancement ratio, which compares the tumor’s enhancement to that of the back muscle, varied across patients; this ranged from 1.16 to 3.03, with an average value of 1.73 (with a standard deviation of 0.63). This ratio helped to classify the tumor’s enhancement as either good or moderate, with four patients in each category, as illustrated in Figure 2.

Of particular note was the observation of necrosis within the tumor in five of the eight patients. However, none of the patients in this study were found to have malignant bowel obstruction, a condition characterized by the blockage of the bowel due to cancer. These comprehensive data, which combine both clinical presentations and detailed imaging findings, provide a rich source of information for understanding the characteristics of these colorectal tumors.

**Figure 4:**
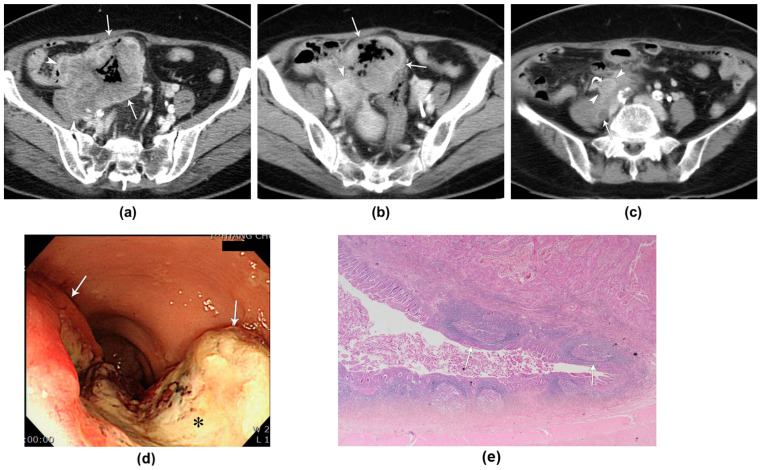
A 70-year-old woman with squamous-cell carcinoma of the sigmoid colon. (**a**,**b**) Contrast-enhanced portal venous phase computed tomography (CT) images demonstrate a peripheral well-enhanced bulky mass (arrows) with internal necrosis in the sigmoid colon. Note the aggressive tumoral invasion (arrowheads) into the adjacent small bowel, right psoas muscle, right ovary, and right ureter (not shown in this figure). (**c**) The contrast-enhanced portal venous phase CT image depicts a large metastatic lymph node (arrowheads) with focal necrosis (curved arrow) along the right common iliac vessel and a dilated right ureter (arrow) due to tumoral invasion. (**d**) A colonoscopic image shows a large ulcerofungating mass (arrows) covered with whitish exudates and tissue debris (asterisk). (**e**) A photograph of the pathological specimen shows the solid nests (arrows) of squamous epithelial cells infiltrating from the mucosa to the muscle layer (low magnification, 20×; hematoxylin–eosin staining).

The total count of metastatic lymph nodes in each patient varied widely, ranging from 0 to as many as 10 nodes. On average, the number of metastatic lymph nodes was 3.12, accompanied by a standard deviation of 3.28; this indicated a considerable variation among patients. Additionally, the size of these lymph nodes, which was measured using the short diameter, ranged from 8 mm to 25 mm; they had an average diameter of 16.57 mm and a standard deviation of 5.68 mm. A notable observation was the presence of necrosis within the metastatic lymphadenopathies in a majority of the cases—specifically, six out of seven patients. The presence of necrosis within lymph nodes is a significant indicator that the disease is aggressive and at an advanced stage. Furthermore, the invasion of adjacent organs was identified in five of the eight patients. This high incidence of organ invasion highlights the aggressive nature of the disease in these patients (Figure 4). Interestingly, distant metastases to the liver were found in one of the eight patients. These hepatic metastases manifested as multiple masses in the liver that appeared as low- or iso-attenuating, with peripheral enhancement during the portal venous phase of the CT scan. 

Two patients underwent additional abdominal ultrasound examinations, which provided further insights. In one patient, the US images revealed target-like peripheral hyperechoic hepatic metastases. In another patient, right hydronephrosis was detected; this was caused by the invasion of the ipsilateral ureter by the primary tumor. 

18F-FDG PET-CT scans were performed on two patients. These scans revealed the presence of hypermetabolic rectal masses, indicating that there was high metabolic activity within the tumor. Additionally, both regional and distant hypermetabolic metastatic lymph nodes were observed in these patients. Hypermetabolic metastases were also found in one patient with multiple hepatic metastases. The use of 18F-FDG PET-CT in these patients provided crucial information regarding the metabolic activity of the tumors and the extent of metastasis, complementing the findings obtained from the CT scans and ultrasound.

## 4. Discussion

The clinical manifestations of primary colorectal SCC often mirror those observed in cases of colon adenocarcinoma. These similarities include a range of symptoms such as hematochezia, changes in bowel habits, abdominal pain, weight loss, and anorexia, as noted in previous studies [17]. In the context of our study, a significant majority of the patients, specifically seven out of eight, presented with symptoms predominantly characterized by hematochezia and abdominal pain. This finding is indicative of the symptomatic parallels between primary colorectal SCC and more common types of colorectal cancer, such as adenocarcinoma.

In further accordance with the established medical literature [18], the rectum and the proximal colon are commonly reported as the sites most frequently affected by primary colorectal SCC. This pattern was largely reflected in the cohort of our study, with all but one case of primary colorectal SCC being located in the rectum; this case presented with involved primary colorectal SCC originating from the sigmoid colon. This distribution underscores the predilection of primary colorectal SCC for the rectal region, and also highlights the possibility of its occurrence in other segments of the colon, such as the sigmoid.

The infrequency of primary colorectal SCC has resulted in a limited understanding of its natural history and characteristic imaging findings. Despite the small number of primary colorectal SCC cases presented in this study, distinct CT imaging features were observed; this suggests that specific radiological patterns are associated with this cancer type. The cases of primary colorectal SCC typically presented as either marked wall thickening or as a bulky mass; this was often accompanied by heterogeneous enhancement that was either well or moderately defined in the involved colonic wall. These tumors were also associated with multiple large lymph nodes. The degree of enhancement seen in primary colorectal SCC on CT scans tended to be slightly more intense than or similar to that observed in adenocarcinoma. Features such the tumor morphology, the degree of enhancement, and the characteristics of metastatic lymph nodes may help to differentiate primary colorectal SCC from the more common colonic adenocarcinoma in CT imaging. However, further research on this is needed.

Regarding tumor necrosis and lymph node involvement, primary colorectal SCC generally exhibited a necrotic nature, which is in contrast to colonic lymphoma. In cervical metastatic lymph nodes that have been confirmed pathologically in patients with oral SCC, contrast-enhanced CT often revealed central low-attenuated areas or heterogeneous densities; these are indicative of tumor infiltration, including tumor necrosis and the keratinization of tumor cells [19]. Furthermore, the rim enhancement that was observed in central necrotic lymph nodes was attributable to the dilation of blood vessels in the lymph node capsule. In our study, non-enhanced or poorly enhanced areas within the metastatic lymph nodes of primary colorectal SCC were hypothesized to be similar to those observed in oral SCC.

An additional imaging characteristic of primary colorectal SCC that was noted in our study was its apparent aggressive nature and propensity to invade adjacent organs, a finding that might help to differentiate it from lymphoma. This propensity for aggressive invasion, coupled with the distinctive imaging features observed, underlines the need for a nuanced understanding of primary colorectal SCC regarding both its diagnosis and treatment.

One patient from our study presented with hepatic metastases; these manifested as hypovascular lesions characterized by a hyperattenuating rim and a hypoattenuating center during the portal venous phase of imaging. This specific presentation of hepatic metastases is aligned with findings that are commonly associated with squamous-cell carcinomas originating from other organs such as the head and neck, esophagus, and lungs, as documented in previous studies [20,21]. These similarities in imaging characteristics suggest a shared pattern of metastatic behavior among squamous-cell carcinomas, regardless of their primary site.

The staging of primary colorectal SCC is yet to be formalized in the medical community; this has led to the current practice of employing the tumor–node–metastasis (TNM) staging system, which is typically used for colonic adenocarcinoma, to stage primary colorectal SCC [13]. The prognosis for patients with primary colorectal SCC is poorer compared to patients with adenocarcinoma at the same TNM stage [8]. This observation is supported by several previous case reports that have highlighted the tendency of primary colorectal SCCs to be more locally invasive, to exhibit more regional metastatic lymphadenopathies than adenocarcinomas, and to be diagnosed at a later stage [22,23]. These characteristics of primary colorectal SCC, as described in the literature, are in striking concordance with the results of our imaging findings. Moreover, the involvement of lymph nodes in primary colorectal SCC is indicative of a worse prognosis compared to adenocarcinoma at a similar stage [7]. The local invasiveness and frequent nodal involvement that are characteristic of primary colorectal SCC are believed to contribute significantly to its poor prognosis. Interestingly, esophageal SCC exhibits similar patterns, particularly in terms of lymphatic spread. Previous reports have indicated that early-stage esophageal SCC has a higher likelihood of showing lymph node metastasis compared to adenocarcinoma of a similar stage [24]. The presence of lymph node metastases is a significant independent prognostic factor in esophageal malignancies, as is consistently noted in the literature [24,25,26]. Consequently, esophageal SCC, which is akin to primary colorectal SCC, tends to have a poorer long-term prognosis than adenocarcinoma [24]. This parallel between the behaviors of esophageal and primary colorectal SCC underscores the aggressive nature of SCC, regardless of its origin.

To date, the medical community has not established a standardized treatment protocol for primary colorectal SCC. Historically, the primary treatment approach employed for rectal SCC was surgical resection, which could range from localized excisions to more extensive radical surgeries. However, in recent years, there has been a shift in focus towards more integrated treatment strategies. Recent studies have increasingly explored the efficacy of chemoradiation, either as neoadjuvant therapy, which is administered before surgical intervention, or as adjuvant therapy, which is provided after surgery [27]. These approaches have shown promising results and have been associated with encouraging findings in the field of oncology, particularly in the past decade [13].

The underlying causes of primary colorectal SCC remain somewhat elusive, but various theories that explain its development have been proposed. Among these, the theory positing that chronic inflammation of the colonic mucosal epithelium leads to squamous metaplasia, followed by carcinogenesis, is the most persuasive. This hypothesis is supported by the identification of several risk factors associated with chronic inflammation, which may contribute to the development of squamous metaplasia. These factors include ulcerative colitis [28], infections such as schistosomiasis [29], amebiasis [14], and human papillomavirus infection [30], and exposure to radiation [31]. Another intriguing hypothesis posits the squamous differentiation of adenomas and adenocarcinomas. This idea has been suggested in various studies, which have reported the occurrence of squamous metaplasia in adenomatous polyps [32,33,34,35], and cases in which SCC has arisen from villous adenoma [18]. These theories and findings contribute to a growing understanding of the complex etiology of primary colorectal SCC, and indicate a multifactorial origin involving both chronic inflammatory processes and potential squamous differentiation.

Our study has several limitations. Firstly, the small sample size is a significant constraint, and can primarily be attributed to the rarity of primary colorectal SCC. Additionally, the retrospective nature of this study inherently imposes certain limitations, such as potential biases and constraints regarding data availability. Secondly, there is notable heterogeneity in the CT scanning parameters used in this study. This includes the employment of various CT scanners and differing scanning protocols, which is again a byproduct of the infrequency of this disease. Despite this variability in the CT scanning process, we maintain that the quality of the CT images obtained was sufficiently high; this enabled an effective evaluation of the CT findings pertinent to primary colorectal SCC. A third limitation of our study is that not all instances of tumor necrosis, nodal involvement, and distant metastasis were verified through pathological examination. However, it is noteworthy that the instances of nodal involvement and distant metastasis in primary colorectal SCC, which were not pathologically confirmed, were clinically validated. This clinical confirmation was based on the observation of tumor growth in follow-up CT imaging, particularly in the two patients who underwent palliative surgery. Despite these efforts, a precise point-to-point radiologic–pathologic correlation could not be established. Nevertheless, despite these limitations, our study has revealed that there are consistent imaging patterns associated with primary colorectal SCC. These patterns could enhance our understanding of this rare disease and may contribute to improved diagnostic accuracy and treatment strategies in the future.

## 5. Conclusions

In the realm of gastroenterological oncology, primary colorectal SCCs are notably rare entities. However, our research has shown that, despite their rarity, these carcinomas exhibit distinct and characteristic findings on CT scans. Typically, primary colorectal SCC presents as aggressive and invasive, and is often characterized by marked wall thickening or the formation of a bulky mass. These tumors frequently show heterogeneous and well-defined enhancement patterns on CT scans, coupled with internal necrosis. Another significant aspect observed in these cases is the frequent occurrence of nodal involvement, with the nodes tending to be large in size and often exhibiting necrotic features. Additionally, a common radiologic feature of primary colorectal SCC is its tendency to invade adjacent structures.

The identification of these characteristic CT findings may play a crucial role in both the diagnosis of primary colorectal SCC and the development of treatment strategies for this rare type of cancer. Furthermore, these imaging characteristics are pivotal in predicting the clinical prognosis of patients afflicted with primary colorectal SCC. Thus, our study underscores the importance of recognizing these specific radiological patterns; this may aid clinicians in navigating the complexities associated with this uncommon but aggressive form of colorectal cancer.

## Figures and Tables

**Table 1:** Clinical and imaging findings of eight patients with primary squamous-cell carcinoma of the colon.

Age (y)/ Sex	Clinical Presentation	Radiologic Studies	Tumor Location	Morphologic Type	Maximum Wall Thickness (mm)	Enhancement Pattern of the Tumor *	Enhancement Degree ^†^	Tumor Necrosis	LAP (Number/Diameter (mm)) ^‡^	LAP Necrosis	Adjacent Organ Invasion	Distant Metastases
70/F	Abdominal pain	CT, US	Sigmoid colon	Bulky mass	44	Heterogeneous	Well (3.01)	Yes	0/NA	NA	Right ureter and ovary, ileum, urinary bladder, iliopsoas muscle, and uterus	No
59/M	Hematochezia	CT, PET-CT	Rb	Circumferential wall thickening	24	Homogeneous	Moderate (1.22)	No	10/21	Yes	Levator ani muscle, prostate, urinary bladder	No
67/F	Hematochezia	CT	Ra	Eccentric wall thickening	14	Homogeneous	Moderate (1.36)	Yes	2/12	Yes	No	No
92/M	Hematochezia, constipation	CT	Rb	Bulky mass	44	Heterogeneous	Well (1.53)	Yes	2/17	Yes	Prostate	No
54/F	Fever	CT, US, PET-CT	Ra	Bulky mass	49	Heterogeneous	Well (2.02)	Yes	8/21	Yes	Uterus	Liver
86/M	Hematochezia	CT	Rb	Circumferential wall thickening	25	Homogeneous	Well (2.36)	No	1/25	Yes	Levator ani muscle	No
76/M	Abdominal pain	CT	Ra	Circumferential wall thickening	14	Homogeneous	Moderate (1.23)	No	1/8	No	No	No
54/F	Hematochezia	CT	Rb	Circumferential wall thickening	19	Homogeneous	Moderate (1.16)	Yes	1/12	Yes	No	No

* The tumor is defined as the non-necrotic enhancing portion within the tumor, excluding the area of necrosis. ^†^ The tumor-to-back-muscle enhancement ratio on the portal venous phase image of the contrast-enhanced CT. Good, moderate, and poor tumor enhancement are defined as a tumor-to-back-muscle enhancement ratio of >1.5, 1–1.5, and <1, respectively, on CT. ^‡^ LAP (number/diameter (mm)) indicates lymphadenopathy (number of lymphadenopathy/short diameter of largest lymph node (mm)). Abbreviations: LAP = lymphadenopathy; NA = not applicable; Ra = rectum above the peritoneal reflection; Rb = rectum below the peritoneal reflection.

## Data Availability

The data presented in this study are available upon request from the corresponding author. The data are not publicly available for confidentiality reasons.

## References

[1] Sung H., Ferlay J., Siegel R.L., Laversanne M., Soerjomataram I., Jemal A., Bray F. (2021). Global cancer statistics 2020: GLOBOCAN estimates of incidence and mortality worldwide for 36 cancers in 185 countries. CA Cancer J. Clin..

[2] Aung Z.W., Carina M.A. (2015). A case of metastatic rectal squamous cell carcinoma initially diagnosed as lung cancer. J. Clin. Imaging Sci..

[3] Neil V., Sumair A., Khaled B., Carmine C., Hassan A., Robert S., James N., Aaron W., Joshua A. (2016). Primary squamous cell carcinoma of the rectum: A case report and literature review. J. Community Hosp. Intern. Med. Perspect..

[4] Pikarsky A.J., Belin B., Efron J., Woodhouse S., Weiss E.G., Wexner S.D., Nogueras J.J. (2007). Squamous cell carcinoma of the rectum in ulcerative colitis: Case report and review of the literature. Int. J. Colorectal. Dis..

[5] Gelas T., Peyrat P., Francois Y., Gerard J.P., Baulieux J., Gilly F.N., Vignal J., Glehen O. (2002). Primary squamous-cell carcinoma of the rectum: Report of six cases and review of the literature. Dis. Colon Rectum.

[6] Musio D., De Felice F., Manfrida S., Balducci M., Meldolesi E., Gravina G.L., Tombolini V., Valentini V. (2015). Squamous cell carcinoma of the rectum: The treatment paradigm. Eur. J. Surg. Oncol..

[7] Frizelle F.A., Hobday K.S., Batts K.P., Nelson H. (2001). Adenosquamous and squamous carcinoma of the colon and upper rectum: A clinical and histopathologic study. Dis. Colon Rectum.

[8] Comer T.P., Beahrs O.H., Dockerty M.B. (1971). Primary squamous cell carcinoma and adenocanthoma of the colon. Cancer.

[9] Bartelink H., Roelofsen F., Eschwege F., Rougier P., Bosset J.F., Gonzalez D.G., Peiffert D., van Glabbeke M., Pierart M. (1997). Concomitant radiotherapy and chemotherapy is superior to radiotherapy alone in the treatment of locally advanced anal cancer: Results of a phase III randomized trial of the European Organization for Research and Treatment of Cancer Radiotherapy and Gastrointestinal Cooperative Groups. J. Clin. Oncol..

[10] Jaffer A.A., Kathryn A.W., Leonard L.G., Pedersen J., Benson A.B., Thomas C.R., Mayer R.J., Haddock M.G., Rich T.A., Willett C. (2008). Fluorouracil, mitomycin, and and radiotherapy vs fluorouracil, cisplantin, and radiotherapy for carcinoma of the anal canal: A randomized controlled trial. JAMA.

[11] Al B.B., Alan P.V., Mahmoud M.A.H., Azad N., Chen Y.-J., Ciombor K.K., Cohen S., Cooper H.S., Deming D., Garrido-Laguna I. (2023). Anal carcinoma, version 2.2023, NCCN clinical practice guidelines in oncology. J. Natl. Compr. Cancer Netw..

[12] Nahas C.S., Shia J., Joseph R., Schrag D., Minsky B.D., Weiser M.R., Guillem J.G., Paty P.B., Klimstra D.S., Tang L.H. (2007). Squamous-cell carcinoma of the rectum: A rare but curable tumor. Dis. Colon Rectum.

[13] Guerra G.R., Kong C.H., Warrier S.K., Lynch A.C., Heriot A.G., Ngan S.Y. (2016). Primary squamous cell carcinoma of the rectum: An update and implications for treatment. World, J. Gastrointest. Surg..

[14] Williams G.T., Blackshaw A.J., Morson B.C. (1979). Squamous carcinoma of the colorectum and its genesis. J. Pathol..

[15] Macari M., Balthazar E.J. (2001). CT of bowel wall thickening: Significance and pitfalls of interpretation. Am. J. Roentgenol..

[16] Anthony T., Baron T., Mercadante S., Green S., Chi D., Cunningham J., Herbst A., Smart E., Krouse R.S. (2007). Report of the clinical protocol committee: Development of randomized trials for malignant bowel obstruction. J. Pain Symptom Manag..

[17] Lafreniere R., Ketcham A.S. (1985). Primary squamous carcinoma of the rectum. Report of a case and review of the literature. Dis. Colon Rectum.

[18] Lundquest D.E., Marcus J.N., Thorson A.G., Massop D. (1988). Primary squamous cell carcinoma of the colon arising in a villous adenoma. Hum. Pathol..

[19] Morimoto Y., Kurokawa H., Tanaka T., Yamashita Y., Kito S., Okabe S., Takahashi T., Ohba T. (2006). Correlation between the incidence of central nodal necrosis in cervical lymph node metastasis and the extent of differentiation in oral squamous cell carcinoma. Dentomaxillofac. Radiol..

[20] Sica G.T., Ji H., Ros P.R. (2000). CT and MR imaging of hepatic metastases. Am. J. Roentgenol..

[21] Semelka R.C., Helmberger T.K. (2001). Contrast agents for MR imaging of the liver. Radiology.

[22] Lasser P., Elias D., Eschwege F. (1980). Epidermoid epitheliomas of the rectum: A report on three cases. J. Chir..

[23] Miyamoto K., Nishioka M., Kurita N., Honda J., Yoshikawa K., Higashijima J., Miyatani T., Bandou Y., Shimada M. (2007). Squamous cell carcinoma of the descending colon: Report of a case and literature review. Case Rep. Gastroenterol..

[24] Siewert J.R., Stein H.J., Feith M., Bruecher B.L., Bartels H., Fink U. (2001). Histologic tumor type is an independent prognostic parameter in esophageal cancer: Lessons from more than 1,000 consecutive resections at a single center in the western world. Ann. Surg..

[25] Akiyama H., Tsurumaru M., Udagawa H., Kajiyama Y. (1994). Radical lymph node dissection for cancer of the thoracic esophagus. Ann. Surg..

[26] Ando N., Ozawa S., Kitagawa Y., Shinozawa Y., Kitajima M. (2000). Improvement in the results of surgical treatment of advanced squamous esophageal carcinoma during 15 consecutive years. Ann. Surg..

[27] Rasheed S., Yap T., Zia A., McDonald P.J., Glynne-Jones R. (2009). Chemo-radiotherapy: An alternative to surgery for squamous cell carcinoma of the rectum--report of six patients and literature review. Color. Dis..

[28] Cheng H., Sitrin M.D., Satchidanand S.K., Novak J.M. (2007). Colonic squamous cell carcinoma in ulcerative colitis: Report of a case and review of the literature. Can. J. Gastroenterol..

[29] Wiener M.F., Polayes S.H., Yidi R. (1962). Squamous carcinoma with schistosomiasis of the colon. Am. J. Gastroenterol..

[30] Sotlar K., Koveker G., Aepinus C., Selinka H.C., Kandolf R., Bultmann B. (2001). Human papillomavirus type 16-associated primary squamous cell carcinoma of the rectum. Gastroenterology.

[31] Yurdakul G., de Reijke T.M., Blank L.E., Rauws E.A. (2003). Rectal squamous cell carcinoma 11 years after brachytherapy for carcinoma of the prostate. J. Urol..

[32] Chen K.T. (1981). Colonic adenomatous polyp with focal squamous metaplasia. Hum. Pathol..

[33] Almagro U.A., Pintar K., Zellmer R.B. (1984). Squamous metaplasia in colorectal polyps. Cancer.

[34] Forouhar F. (1984). Neoplastic colonic polyp with extensive squamous metaplasia. Tumori J..

[35] Kontozoglou T. (1985). Squamous metaplasia in colonic adenomata: Report of two cases. J. Surg. Oncol..

